# An Analysis of an Inventory of Community Resilience Frameworks

**DOI:** 10.6028/jres.126.031

**Published:** 2021-10-20

**Authors:** Jarrod Loerzel, Maria Dillard

**Affiliations:** 1 National Institute of Standards and Technology, Gaithersburg, MD 20899, USA

**Keywords:** assessment methodology, community resilience, indicators, resilience frameworks

## 1 Summary

While resilience is a concept that has many definitions [1-8], the National Institute of Standards and Technology (NIST) and other federal agencies have adopted the definition of resilience as proposed in the 2013 Presidential Policy Directive 21 which states that resilience is "the ability to prepare for anticipated hazards, adapt to changing conditions, and withstand and recover rapidly from disruptions" [8]. However, a missing accompanying element to this definition is the way in which resilience is to be measured. In response, there is a growing field of research focused on community-level resilience; academic, governmental, and private sector researchers have developed and examined approaches to measure the concept both quantitatively and qualitatively.

As a result, frameworks have been developed to connect concepts of resilience to measurable indicators and/or measures to operationalize the concept of resilience. They have emerged both as a methodology to study community resilience and as a decision support tool for disaster and adaptation planning. However, reviews by the NIST Community Resilience Program [9-12] and others [13-16] have shown that there is a lack of consensus in terms of the theoretical approaches taken, indicators and measures used, data requirements, and spatial scales among the frameworks. To better understand these disparities, NIST constructed an inventory of resilience frameworks.

This data article provides details of an analysis of an inventory of 56 community resilience frameworks. In this article, a description of how the inventory data was catalogued is presented. Section 3 outlines the categories used to analyze the frameworks as well as the categories used to bin the indicators and measures used in each. Section 3.1 describes the results of the categorization process for the 56 resilience frameworks. Section 3.2 outlines the results of the categorization of the 3,298 *indicators* used in each framework. Section 3.3 describes the 7,165 *measures* used in the 56 frameworks.

**2:** Data Specifications

**NIST Operating Unit(s)**	Engineering Laboratory, Materials and Structural Systems Division
**Format**	Tabular data file (.xls)
**Instrument**	N/A
**Spatial or Temporal Elements**	N/A
**Data Dictionary**	https://data.nist.gov/od/id/mds2-2297
**Accessibility**	All datasets submitted to *Journal of Research of NIST* are publicly available.
**License**	https://www.nist.gov/director/licensing
	

## 3 Methods

A sample of 56 community resilience frameworks and assessment methods was gathered by subject matter experts in the areas of community resilience and indicator-based measurement. The frameworks cover a variety of disciplinary perspectives (*e.g.,* economics, urban planning, and engineering) and numerous focal areas (*e.g.,* natural, physical, and/or social systems). Further, these frameworks operate at different spatial scales, are aimed at a variety of audiences, and are at various stages of development and implementation (from those that are entirely conceptual to those that have already been applied). This sample of community resilience frameworks is not an exhaustive list of every resilience indicator or resilience assessment methodology. However, it provides breadth of coverage of units of analysis, hazards, and approaches in the field.

The data entry began by examining the 56 resilience frameworks and categorizing the focal area (or areas) contained within them. Broad, general categories were used rather than a more focused approach to catalog the themes of each framework because this approach enables the comparison of frameworks against each other and it captures the variety of indicators used within each of the 56 frameworks. The focal areas were identified based on a review of each framework and its constituent indicators. The frameworks were then cataloged into seven overarching focal areas, as well as a category for *Not clear* where no focal area was identified (Table 1).

**Table 1:** Definitions of focal areas.

Framework focus	Definition
Buildings	Individual building specific
Infrastructure	Infrastructure systems of any type
Economic	Finances, funding, poverty, etc.
Health	Public health, disease, illness, nutrition, access to medical care, etc.
Other social	Welfare, politics, social connectedness, community collaboration, etc.
Natural	Environment, ecosystems, land, animals, etc.
Other	Focal areas other than those listed above
Not clear	Focal area is unclear or cannot be determined based on the framework text

In the preliminary assessment, *Buildings* and *Infrastructure* categories were combined, but it was later determined that the categories needed to be split because some frameworks were focused on infrastructure systems writ large (and included buildings), some were applicable only to buildings. The broad *Economic* category included everything from public financing, government expenditures, poverty related issues, or other economic concerns. Thus, if the framework had any mention of these economic issues as an indicator focal area, it was included. Likewise, frameworks that touched on any area of population health were included in our *Health* category which encompassed focal areas of public health, disease or illness rates, ensuring adequate nutrition, and access to medical care, among others. The *Other social* category was used to bin frameworks that included broad, difficult to measure concepts of social interaction and political dynamics. These included mentions of concepts such as social connectedness, social cohesion, community collaboration activities, and participation in elections. The amount of arable land, animal ownership and husbandry techniques, ecosystem condition, and overall environmental status were components of the *Natural* category. Frameworks could be categorized by more than one focal area. Any mention of a focal area not captured in any of our defined categories were binned in the *Other* category. Similarly, if the focal area(s) of a framework were not addressed in the documentation describing it, then it was categorized as *Not clear*.

Twenty-two categories were developed to catalog the unit of analysis used by each of the 56 frameworks in their resilience assessment. The final units of analysis are detailed in Table 2.

**Table 2:** Units of analysis.

Individual	Municipality
Household	County
Building	Metropolitan area
Organization	Intrastate region
Tribe	State / territory
Parcel	Interstate region
Census block	Country
Census block group	Global
Census tract	Ecosystem
ZIP code	Other
Community	Not provided

The resilience frameworks were also categorized by the hazard type(s) to which they were applicable. Fourteen hazard types were found within the documentation of each framework. The hazards were: climate change/sea level rise; drought; earthquake; three different types of flooding (inland, tsunami, and wind-driven surge); hurricane; tornado; wildfire; winter storm; other natural hazards; disease; technological or human-caused hazards; and, terrorism. Frameworks that are not designed to a specific hazard are also included.

Each framework was examined to determine if it had been utilized in a real-world application or was only conceptual in nature. This category was named *Status*, and each framework was binned accordingly. A *Not clear* option was also included for those frameworks where there was not enough information to classify it appropriately.

*Indicators* use quantitative or qualitative data to establish the relative value of a given property in a specific community system, and, if the data is collected over a period of time, indicators can shed light on any direction of change from the initial status [17]. Indicators are often used in combination to create a composite indicator. Composite indicators utilize a variety of indicators coupled with various mathematical methods to arrive at a single value representing a complex relationship [18]. As such, each indicator was categorized according to how they are constructed: it was comprised of multiple measures (a composite indicator) or the indicator consisted of a single measure. The unit of analysis was evaluated for each indicator along with whether the indicator was an *input* or was an *output* in the assessment of community resilience. For example, *input* was assigned when the framework specified that the indicator is used to contribute to the development of an overall score, and *output* was selected when the framework specified that the indicator is the overall score that is derived from the measurement.

*Measures* are the data used to operationalize the theoretical indicators of the various frameworks. The measures used by the 56 frameworks were characterized in several ways. First, the measures were cataloged across the frameworks in relation to the associated indicator. Then, for each measure, the following information was documented: units of analysis, spatial reporting (i.e., the spatial area for which the measure is gathered), temporal reporting (i.e., how often the measure data is collected), and availability (i.e., if the measure had been collected or if a new collection effort would be required). Additionally, the measure type was recorded (i.e., if the measure was binary, categorical, continuous, etc.), the measure’s availability and source location (i.e., if available, where the data can be found), and, if the measure was ultimately transformed into a score of resilience in the framework.

The data entered in the inventory was analyzed using basic descriptive statistics. Section 3.1 describes the results of the framework categorization, Sec. 3.2 describes the results of the indicator categorization, and Sec. 3.3 describes the results of the measure categorization.

### 3.1 Description of the frameworks

**Fig. 1:**
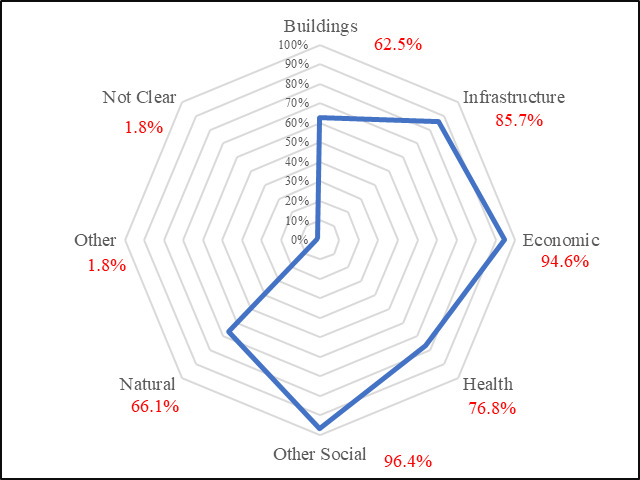
Focal areas of frameworks.

The results show that most frameworks include a focus on indicators of *Other social* and *Economic*: 96.4% (n=54) and 94.6% (n=53), respectively. The concentration of indicators in the *Other social* category is evidence of the reliance on social system indicators when measuring community resilience. When compared to categories such as *Buildings* or *Natural*, a social focus was more consistently employed across frameworks. An *Infrastructure* focus was included in 85.7% (n=48) of the frameworks (Fig. 1).

**Fig. 2:**
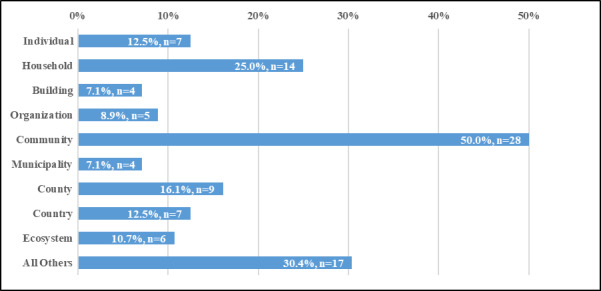
Units of analysis of frameworks.

Many of the frameworks spanned multiple units of analysis. However, *Community* and *Household* units of analysis were the most frequently used by the frameworks, with 50.0% (n=28) and 25.0% (n=14), respectively (Fig. 2). The *County* unit of analysis was also commonly used, with a frequency of 16.1% (n=9). No frameworks assessed resilience at the *Parcel* level. Similarly, no frameworks used the *US Census block group* or *ZIP code* as a unit of analysis. Unsurprisingly, no frameworks attempted to use a *Global* unit of analysis. The unit of analysis was not provided in 5.4% (n=3) of the frameworks (Fig. 2 and Table 3).

**Table 3:** The units of analysis for the frameworks.

Counts of units of analysis No. (% of frameworks utilizing the unit)				
Community	28 (50.0%)		State/Territory	3 (5.4%)
Household	14 (25.0%)		Not provided	3 (5.4%)
County	9 (16.1%)		Tribe	2 (3.6%)
Country	7 (12.5%)		Census tract	1 (1.8%)
Individual	7 (12.5%)		Census block	1 (1.8%)
Ecosystem	6 (10.7%)		Interstate region	1 (1.8%)
Organization	5 (8.9%)		Census block group	0 (0%)
Building	4 (7.1%)		Parcel	0 (0%)
Municipality	4 (7.1%)		ZIP code	0 (0%)
Metropolitan area	3 (5.4%)		Global	0 (0%)
Intrastate region	3 (5.4%)		Other	0 (0%)

**Fig. 3:**
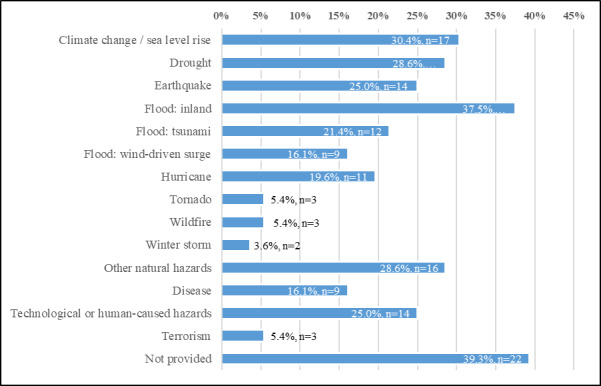
Hazards of interest in frameworks.

Figure 3, below, shows that of the 56 frameworks analyzed, 60.7% (n=34) of the frameworks were applicable to specific hazard events. Many of the frameworks, 37.5% (n=21), focused on *Inland flood* events and 30.4% (n=17) focused on *Climate change* and/or *Sea level rise*. Approximately twenty-eight percent of the frameworks could be or were applied to the resiliency of *Drought* events (n=16) as well as to *Other Natural hazards* (n=16). The categories of *Earthquake* and *Technological or human-caused hazards* were the focus of 25.0% (n=14, each) of the frameworks.

*Tsunamis* and *hurricanes* were the hazard focus of a smaller proportion of the frameworks in our analysis, at 21.4% and 19.3%, respectively. *Wind-driven surge* and *Disease* each had 16.1% (n=9, each) of the hazard focus in the frameworks, while 5.4% (n=3, each) of the frameworks focused on *Tornadoes*, *Wildfire*, or *Terrorism*, individually. And *Winter storms* were applicable to 3.6% (n=2) of the frameworks.

Most of the frameworks, 64.3% (n=36), were conceptual (i.e., not yet implemented) while 18 (32.1%) were implemented at some point. Two frameworks had unique categorization: (1) Cutter’s *Social Vulnerability Index* (*SoVI*)^©^ was deemed both conceptual and implemented because the source material includes both a conceptual framework as well as an operational version that has been implemented, and (2) *IASC In-Country Team Self-Assessment Tool for Natural Disaster Response Preparedness*, where the status could not be determined from the framework’s literature (Table 4)^1^1 The researchers decided to not go beyond the primary literature when populating the Inventory..

**Table 4:** Framework status.

Status	Count
Conceptual	36 (64.3%)
Implemented	18 (32.1%)
Conceptual/implemented	1 (1.8%)
Not clear	1 (1.8%)

**Fig. 4:**
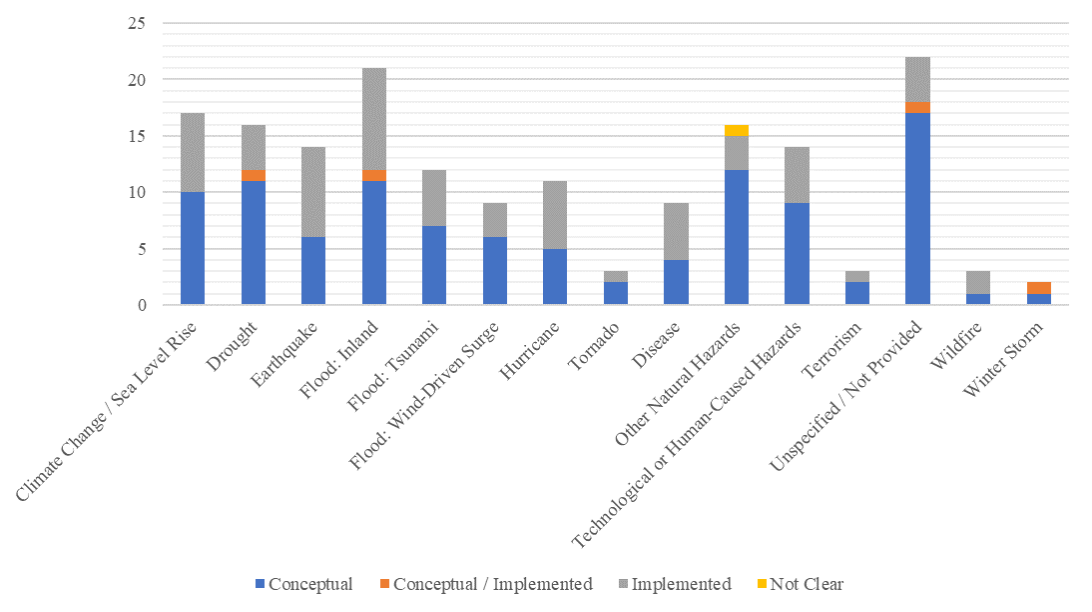
Crosstabulation results of framework status by hazard focus.

When considering the status of the frameworks in relation to the hazard focus, Fig.4 shows that for only three hazards did implemented frameworks outnumber conceptual frameworks: earthquakes, hurricanes, and disease. Eight implemented frameworks, compared to six conceptual frameworks, used an earthquake hazard scenario to develop resilience assessments. The counts of implemented and conceptual frameworks for hurricane and disease focused resilience assessments are closer. Six implemented frameworks and five conceptual frameworks used a hurricane scenario for resilience assessment. And five implemented frameworks and four conceptual frameworks were designed for a disease hazard context. The same pattern continues with inland flooding hazards where 11 resilience assessment frameworks were categorized as conceptual, 9 as implemented, and one as both conceptual and implemented. Finally, of the total of 17 frameworks developed for assessments of resilience to climate change and/or sea level rise, 10 were categorized as conceptual and 7 as implemented frameworks.

**Fig. 5:**
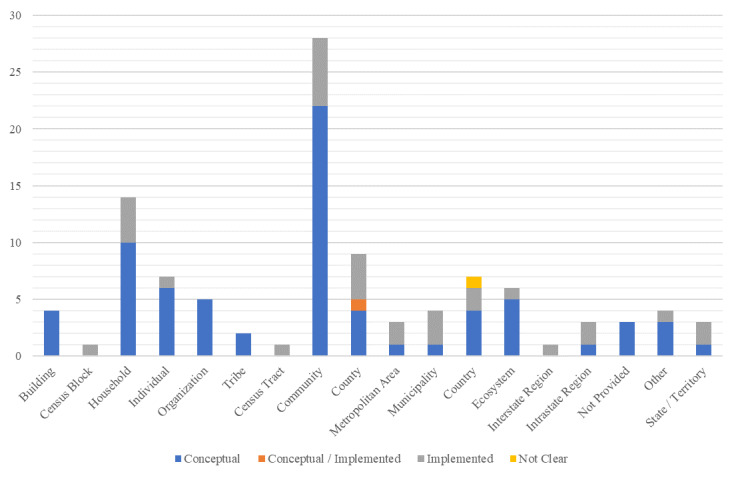
Crosstabulation results of framework status by unit of analysis.

Figure 5 shows that there were six units of analysis where implemented frameworks outnumbered conceptual frameworks: the Census block, metropolitan area, municipality, interstate region, intrastate region, and state/territory. Most of the conceptual frameworks used the community as a spatial unit of analysis (n=22) which was almost four times higher than the six frameworks that have been implemented used at this level of analysis. The next most used unit of analysis was the household, where 10 conceptual frameworks and 4 implemented frameworks estimated resilience at this level.

### 3.2 Description of the indicators

Looking at the single-measure indicators, the average number of indicators used was approximately 71 per framework. The highest number of single-measure indicators was recorded for *HAZUS-MH* with 411; the least number was 4 used in the International Institute for Sustainable Development’s Climate Resilience and Food Security (IISD) framework. Composite indicators totaled 669 in the inventory out of a total of 3,298. The most composite indicators were used in the Oregon Resilience Plan at 76, while twelve frameworks contained no composite indicators.

Similarly, the indicator units of analysis consisted of single-measure values and averaged approximately 150 per category. This was based on a total of 3,298 indicators across 22 categories. It should be noted that there was a wide variation in the count per category, ranging from a low of 18 for the *Project Outcome* unit of analysis going up to 1,342 in the *Community* category. The composite indicators totaled 669 across the same 22 units of analysis categories. The *Community* category had the highest number of composite indicators at 192, while no composite indicators were used in three units of analysis categories: *Census Block*, *Census Tract*, and *Census Block Group*.

The highest count of indicators of any type was found in the *HAZUS-MH* framework with 412; the least amount was 5 and found in the 2014 Keating et al. paper titled, “Operationalizing Resilience against Natural Disaster Risk: Opportunities, Barriers, and a Way Forward”, referred to as the *Zurich Flood Resilience Alliance* framework in this analysis.

### 3.3 Description of the measures

There were 7,165 measures used in the 56 frameworks. The *HAZUS-MH* framework utilized the largest number of measures with a total of 2,050; this number included 1,541 measures collected at the *Building* or *Infrastructure component* unit of analysis. The second highest count of measures was within the *Oregon Resilience Plan* which uses 664, all of which were collected at the *Building*, *Infrastructure component*, or the *Infrastructure system* unit of analysis. The fewest measures were found in the *Inter-American Development Bank Disaster Deficit Index* which included 7 single-measure indicators collected at the *Country* level.

There were more measures of *Continuous* variable type than any other (28.1%, n=2,014). And while 1,967 (27.5%) measures could not be associated with a specific variable type or were of unspecified type, the remaining measures could be classified as *Categorical* (20.3%, n=1453), *Integer* (15.7%, n=1124), or *Binary* (8.5%, n=607). There were 1,537 measures that ultimately transformed into a score of resilience and 3,865 that were not used as inputs to a resilience score. However, 1,763 measures were *Not applicable* or *Not specified*, thus it was not known if they could be used as inputs in calculations of resilience scores.

The time interval of the data collected for a given measure was referred to as *Measure Temporal Reporting* in the framework inventory. Some of the measure data sets are collected at routine time intervals (e.g., annually, quarterly, monthly), and other data sets are collected for a single time point. An analysis of the temporal reporting of the measures reveals that over 78% (n=5,601) had no specified or applicable time frame for measure data collection. The second largest number, 1,356 (18.9%), was associated with a “one-time” data collection, which generally refers to survey data collection efforts specific to a project. The third most frequent temporal reporting for measures was on an annual basis (n=80, 1.1%). The remaining data collection periods for the measure reporting are shown in Table 5.

**Table 5:** Measure temporal reporting.

Time interval	Count
5-year average	1
Annual	80
Every 4 years	1
Less freq. than annual	16
Monthly	22
Not applicable	1609
Not specified	3992
One-time	1356
Other	8
Quarterly	18
Semi-annual	5
Variable	57

The geographic unit at which the data for the measure is collected was recorded as *Measure Spatial Reporting* in the inventory. The geographic unit *Community* had 1,449 (20.2%) instances of measure data being collected at that spatial level. *Municipality* was the third most frequently cited spatial unit of measure data (n=724, 10.1%), followed next by *Infrastructure component* at 699 (9.8%). It should be noted that 517, or, 74% of the 699 *Infrastructure component* measure data points come from the *HAZUS-MH* framework. The data for the spatial reporting category was either *Not specified* (n= 166, 2.3%) or *Not applicable* (n=1,494, 20.8%) roughly one fifth of the time. Totals of all measure spatial reporting categories are shown in Table 6.

**Table 6:** Spatial unit reporting for measures.

Spatial unit	Count	Spatial unit	Count
Building	525	Intrastate region	591
Census block	327	Metropolitan area	53
Census tract	224	Multinational	4
Community	1,449	Municipality	724
Country	227	Not applicable	1,494
County	398	Not specified	166
Dataset grid	1	Organization	6
Global	38	Rural community	56
Household	72	State / territory	110
Infrastructure component	699	(blank)	1

The *Measure Unit of Analysis* was documented across all 56 frameworks to describe the geographic unit at which the measure data is collected. There are 51 spatial unit categories from the 7,165 measures recorded. The spatial unit of *Buildings* had a total of 1,157 measures being collected at that scale. It is important to note that 968 (83.6%) originated from one framework: *HAZUS-MH*. The spatial scale *Community* (n=937), followed by *Infrastructure Component* (n=935) ranked very high. However, as with *Buildings*, the *Infrastructure Component* spatial scale was dominated by the *HAZUS-MH* framework with 537, or 61.2% of the total, in that spatial unit. Many measures’ spatial unit was categorized as either *Not Applicable* or *Not Specified* (n=1,694). The remaining results can be found in Table 7.

**Table 7:** Measure unit of analysis.

Measure unit of analysis	Count	Measure unit of analysis	Count
Activities	1	Intrastate region	115
Animal	1	Jobs	1
Animals	1	Jurisdiction	1
Area	4	Livestock source	1
Areas	1	Local economies	1
Associations	1	Local governments	2
Building	1,157	Markets	1
Census block	41	Mechanisms	1
Census tract	30	Metropolitan area	11
Coastal habitats	1	Municipality	670
Colleges	1	Not applicable	1,588
Committees	1	Not specified	106
Community	937	Organization	39
Country	130	Plant	1
County	186	Programs	5
Data	1	Rangeland	2
District	3	Rural community	37
Ecosystem	1	Schools	1
Electrical interruptions	1	Sectors of society	1
Farms	1	Sector	1
Governments	1	Seed source	1
Groups	3	Services	1
Household	250	Social networks	2
Individual	532	State / territory	9
Industry	1	Storms	2
Infrastructure component	935	Tribe	4
Infrastructure system	336	Universities	1
Institutions	1	(blank)	1

The final analysis of measures looked at the availability of the measures. That is, whether the data is already routinely collected and if so, at what scale is the measure data found. A total of 2,780 (38.7%) of the measures require a new collection for each application of the framework as the data is not already available. Among the available measures, 2,399 are collected at the *National* level and 64 are collected at the *International* level. The remaining counts for measure availability can be found in Table 8.

**Table 8:** Measure availability.

Available at:	Count
International	64
National	2,399
National / not specified	7
New collection required	2,780
Non-national government	2
Not applicable	1,568
Not specified	340
Partial	5

## 4 Impact

This report describes the assembly and analysis of an inventory of community resilience indicators and measures, along with their source framework. It represents one of three products to be released as part of the foundational work to develop, analyze, and document existing frameworks for the purpose of developing a community resilience methodology (see the NIST Community Resilience Group products page for more details: link). The inventory is being used by NIST researchers to assess the current state of the field of resilience measurement; it will provide the basis for ongoing analyses to identify consensus in the use of indicators for resilience. If many frameworks use the same indicator or measure, it may indicate a general acceptance in the resilience assessment community as to the utility of that measure or indicator. Alternately, it may indicate that the data are easily collected and therefore, the indicator is less resource intensive. Either way, the indicator can be further tested to examine its properties and value for resilience measurement. NIST researchers envision the inventory as beneficial to academic researchers and practitioners, as well as other community stakeholders, as they work to understand the many community resilience measurement methods available. This report, coupled with other forthcoming products, provides a ready resource for these groups to make use of existing frameworks for tailoring a methodology to assess their community’s resilience, activities such as supporting grant application preparation, or for furthering research in the resilience assessment space.

The inventory has several potential uses and intended audiences. Examples of the types of uses are discussed by audience type below.

Academic researchers may find the inventory useful for further exploring and analyzing the space of resilience measurement. The dataset represents an extensive review in terms of the total number of indicators included and of the dimensions assessed at the framework and indicator/measure levels. Because the attributes are composed of lower-level themes or constructs, researchers will have the opportunity to advance the work by expanding the dimensions included in the evaluation. And because methods utilized in assembling this inventory are extensible across measures and focal areas, new frameworks and methodologies can be added. In fact, researchers are encouraged to expand the number of frameworks within the inventory by downloading and updating the inventory with the entry of new frameworks as they become available. In this way, the inventory will continue to grow and change to support various lines of inquiry related to resilience indicators and assessment methodologies. Likewise, NIST researchers plan to evaluate the need to issue an updated version of the inventory every three years, given the state of the field of community resilience assessment.

Practitioners such as municipal managers, planners, and other government officials can use the data filtering options in the inventory as a tool to identify methodologies that are specifically applicable to the focal area, the needs of the community, the intended use of the assessment, and resources available. For example, by searching for frameworks that will focus on a particular system, at a specific spatial scale, and are geared toward a specific hazard, practitioners can identify options and evaluate the level of technical skill and resources required to implement a particular assessment methodology.

Other community stakeholders may find benefit in use of the specific indicators and measures in the inventory. When applying for grants that require some form of monitoring and evaluation a community representative could search for existing indicators for resilience related characteristics and/or outcomes for which data are already available to meet guidelines. Furthermore, community stakeholders may use the indicator inventory as a source of key performance indicators for a range of plans including floodplain management plans, hazard mitigation plans, and economic development plans, thereby allowing community stakeholders to tailor a methodology to assess their community’s resilience that best suits their needs and specific context.
